# Atypical Orofacial Lupus Vulgaris in a Pediatric Patient: A Report of a Unique Case

**DOI:** 10.7759/cureus.111846

**Published:** 2026-06-30

**Authors:** Hritik V Shah, Aneet Mahendra, Sanjeev Gupta, Aditi Dabhra, Jhanvi Goyal

**Affiliations:** 1 Dermatology, Maharishi Markandeshwar Institute of Medical Sciences and Research, Mullana, IND

**Keywords:** centrofacial plaque, cutaneous tb, extrapulmonary tuberculosis (tb), granulomatous infection, lupus vulgaris, orofacial tuberculosis, pediatric infection

## Abstract

Cutaneous tuberculosis represents only a small fraction of extrapulmonary tuberculosis; however, its consequences can be profoundly disfiguring when diagnosis is delayed. Among its clinical forms, lupus vulgaris is the most common morphological variant, but pediatric involvement of the nasal orifice with extension to the upper lip and intraoral mucosa remains distinctly rare. We report a remarkable case of a nine-year-old male patient with a three-year history of a slowly enlarging erythematous, crusted centrofacial plaque involving the nasal alae, columella, upper lip, and upper gingival mucosa, which was diagnostically challenging due to its unusual site. This case underscores how atypical orofacial lupus vulgaris may masquerade as other chronic granulomatous disorders and highlights the importance of early recognition to prevent progressive tissue destruction, cartilaginous damage, and permanent facial deformity.

## Introduction

Tuberculosis (TB) continues to pose a major global health burden, particularly in developing countries [1]. Cutaneous TB (CTB) is an uncommon form of extrapulmonary TB, accounting for approximately 1-2% of all cases [2-4]. Lupus vulgaris is the most common and progressive form of CTB and typically occurs in individuals with moderate to high cell-mediated immunity to *Mycobacterium tuberculosis* [5-8].

Clinically, lupus vulgaris most frequently involves the head and neck region and may exhibit the characteristic “apple-jelly” appearance on diascopy [9,10]. However, diagnosis becomes especially unusual when lesions are clustered around the nasal orifice with extension onto adjacent mucosal surfaces, particularly in children [3,11]. Such presentations have rarely been described in the literature [4,12].

Oral and intraoral involvement in lupus vulgaris is distinctly uncommon, particularly in children, and may obscure the diagnosis because lesions in this region can mimic other chronic infectious, inflammatory, and granulomatous disorders. In addition, atypical centrofacial distribution with mucosal extension may delay consideration of CTB, especially when bacteriological confirmation is not readily demonstrable in this paucibacillary form. These lesions may persist for years while progressing insidiously, with the potential to cause cartilage destruction and significant deformity if diagnosis is delayed [13,14]. Here, we report an atypical pediatric case of lupus vulgaris involving the nasal orifice, upper lip, and upper gingival mucosa.

## Case presentation

A nine-year-old boy presented to the dermatology outpatient department with a slowly progressive lesion involving the nose and upper lip. The lesion had first appeared three years earlier as a small, pea-sized, red crusted papule over the upper lip and had gradually enlarged to approximately 3 × 4 cm. It remained asymptomatic throughout its course. The onset of the lesion was preceded by a history of chronic rhinorrhea. On further evaluation, the patient gave a history of low-grade intermittent fever for the preceding seven to eight months. There was no history of chronic cough, loss of appetite, or significant weight loss. Notably, a positive contact history for TB was present, as the patient’s grandmother had been diagnosed with TB and had been receiving treatment for the previous four months.

**Figure 1:**
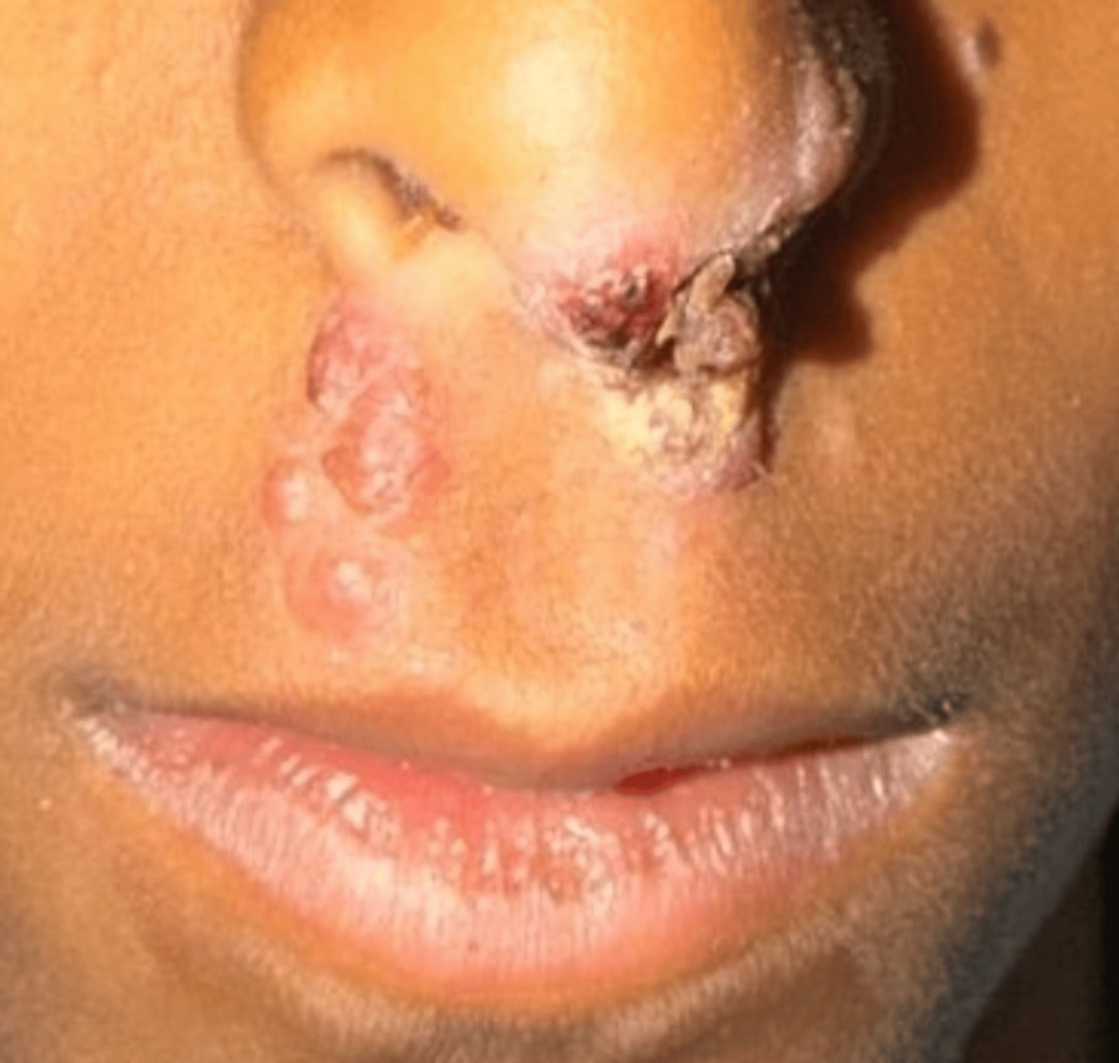
A well-defined erythematous crusted plaque with mild scaling and crusting involving the nasal alae and columella, extending inferiorly to the upper lip, with associated partial loss/distortion of the nasal ala, measuring approximately 3 × 4 cm.

Cutaneous examination showed a well-defined erythematous crusted plaque involving the nasal alae and columella, extending inferiorly to the upper lip, and measuring approximately 3 × 4 cm (Figure 1). Mild scaling and crusting were present over the lesion. There was associated partial loss and distortion of the nasal ala, indicating early tissue destruction and evolving facial deformity. Intraoral examination revealed erythematous swelling of the upper gingival mucosa, an unusual finding that underscored the rare orofacial extension of the disease. Based on the chronicity and morphology of the lesion, differential diagnoses of chronic granulomatous conditions were considered, including CTB (lupus vulgaris), sarcoidosis, and deep fungal infection.

**Figure 2:**
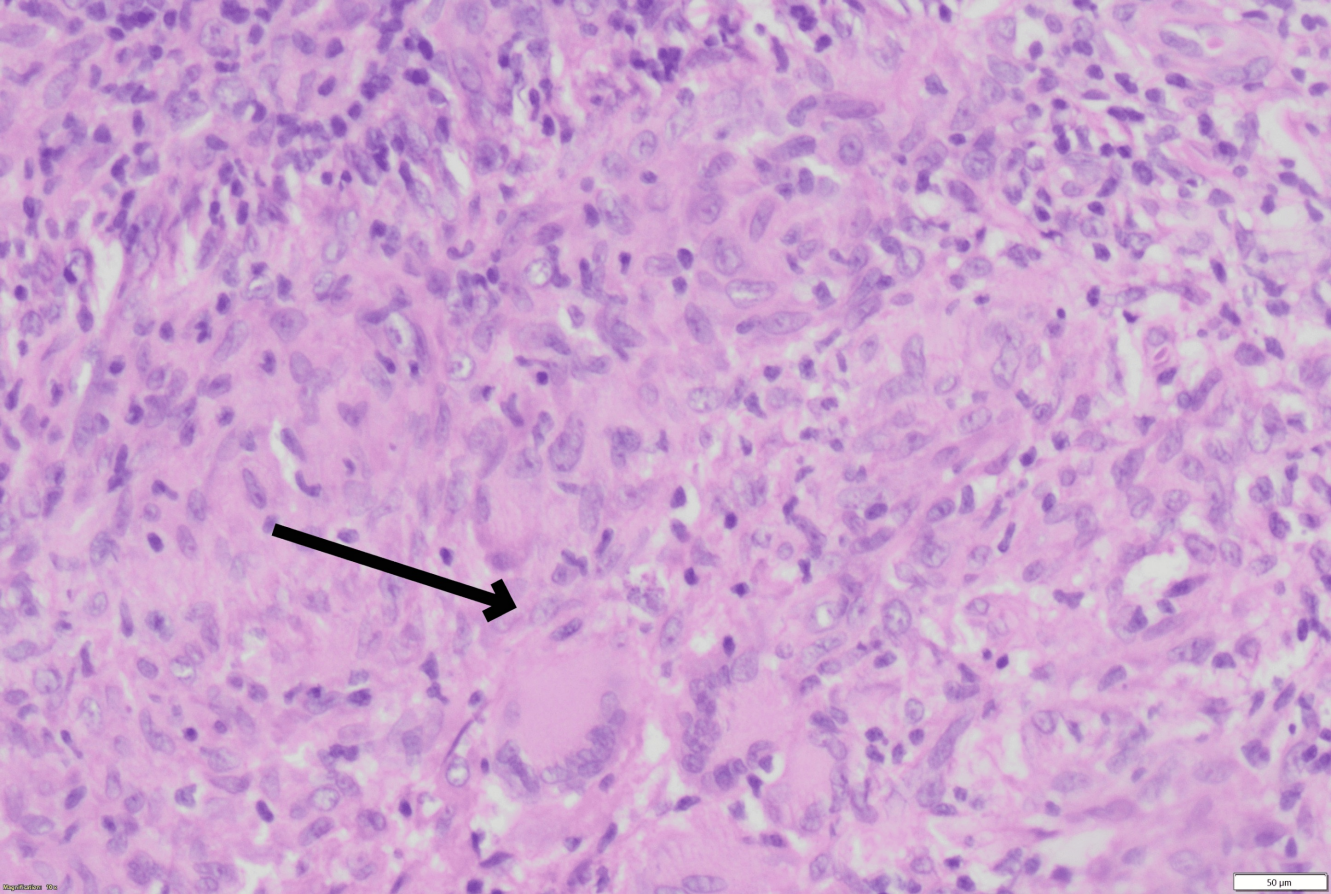
Dermis showing numerous confluent epithelioid cell granulomas with Langhans giant cells (Black arrow), surrounded by dense lymphoplasmacytic infiltrate (Hematoxylin and eosin stain, 20X).

Investigations showed a strongly positive Mantoux test with 27 mm induration, indicating marked hypersensitivity. Histopathological examination of a punch biopsy taken from the upper lip lesion revealed thinning of the overlying epithelium with focal spongiosis. The dermis contained numerous confluent epithelioid cell granulomas with Langhans giant cells, surrounded by dense lymphoplasmacytic infiltrate (Figure 2). The granulomatous process extended into the underlying adipose and muscle tissue. Focal caseous necrosis was noted in occasional granulomas. No dysplasia or malignancy was identified. Special stains were negative for organisms, with Ziehl-Neelsen stain showing no acid-fast bacilli (AFB) and Periodic acid-Schiff with diastase (D-PAS) stain revealing no fungal elements.

Overall, the chronic clinical course, positive contact history for TB, strongly reactive Mantoux test, and histopathological evidence of caseating granulomatous inflammation were considered confirmatory of lupus vulgaris. The patient was advised further clinical correlation with cartridge-based nucleic acid amplification testing (CBNAAT), which was negative, and the patient was referred for initiation of standard anti-tubercular therapy (ATT).

## Discussion

This case is noteworthy not merely because it represents lupus vulgaris in a child, but more importantly because of its unusual site of involvement. Lupus vulgaris is classically described as a chronic, progressive, paucibacillary form of post-primary cutaneous tuberculosis that occurs in individuals with a relatively preserved immune response [5,8]. Although the head and neck are known sites of involvement, predominant localization around the nasal orifice with extension to the upper lip and gingival mucosa is distinctly uncommon, especially in the pediatric age group [3,4,11]. This striking topography broadens the clinical significance of the case, as lesions in this region can closely simulate a variety of inflammatory, granulomatous, and infectious conditions, thereby increasing the risk of diagnostic delay and subsequent deformity.

The pattern of involvement in our patient is clinically noteworthy. The history of chronic rhinorrhea preceding the cutaneous lesion raises the possibility of an initial mucosal focus with contiguous spread to the adjacent skin [8]. Such a sequence offers a plausible explanation for the disease occurring around the nasal orifice and its extension to the upper lip and gingival mucosa. Viewed in this way, the case highlights that lupus vulgaris may not always present as a conventional cutaneous plaque, but may instead evolve across contiguous orofacial surfaces in a deceptive and anatomically unusual manner.

Histopathology was central to establishing the diagnosis. The presence of well-formed tuberculoid granulomas with Langhans giant cells and focal caseous necrosis strongly supports lupus vulgaris, even in the absence of bacteriological confirmation [5]. In this context, the negative Ziehl-Neelsen stain in our case is not contradictory, but rather entirely consistent with the known paucibacillary nature of lupus vulgaris [5,8]. Thus, the biopsy findings assume particular value in atypical presentations such as this one, where the morphology and site may initially suggest a wide range of alternative diagnoses.

The differential diagnosis of destructive or persistent centrofacial granulomatous lesions is broad and includes sarcoidosis, granulomatosis with polyangiitis, and deep fungal infections [12]. However, the histopathological findings in the present case helped to narrow these possibilities in a logical manner. Sarcoidosis typically demonstrates non-caseating granulomas, while granulomatosis with polyangiitis is characterized by necrotizing vasculitis, which was absent in the present biopsy. The negative fungal stain further reduced the likelihood of deep mycosis [4]. Taken together, the chronic course, contact history, Mantoux positivity, characteristic morphology, and supportive histopathology were pivotal in arriving at the diagnosis.

Another important aspect of this case lies in its potential consequences. Despite its indolent progression and the absence of overt systemic decline, lupus vulgaris is not a benign condition. Chronic untreated disease in the nasal region may extend into deeper structures, destroy cartilage, and lead to irreversible deformity, including severe nasal distortion [13,14]. Padmavathy et al. reported a case of ulcerative lupus vulgaris involving the face, highlighting its destructive nature and the potential for delayed diagnosis due to its atypical presentation [15]. Similar to their report, our case underscores the importance of maintaining a high index of suspicion for lupus vulgaris in chronic destructive cutaneous lesions. In a child, such damage carries not only functional implications but also considerable psychosocial consequences. For this reason, the present case is especially significant; it underscores how an uncommon orofacial presentation of lupus vulgaris can mask the diagnosis until late in the disease course. Prompt recognition of this unusual presentation and timely initiation of ATT are therefore essential to arrest progression and prevent permanent facial disfigurement [14].

## Conclusions

This case highlights an atypical and diagnostically challenging presentation of lupus vulgaris involving the nasal orifice, upper lip, and upper gingival mucosa in a child. The rare orofacial distribution, chronic indolent evolution, and negative AFB stain created the potential for delayed recognition. Correlation of clinical history, contact exposure, Mantoux positivity, and histopathological evidence of caseating granulomas was crucial in establishing the diagnosis. The take-home message for clinicians is that they should maintain a high index of suspicion for lupus vulgaris in persistent non-healing centrofacial lesions, particularly in endemic settings. Early diagnosis and prompt initiation of ATT are crucial to prevent cartilage destruction, permanent cosmetic deformity, and long-term morbidity.
